# External Validation of a Case-Mix Adjustment Model for the Standardized Reporting of 30-Day Stroke Mortality Rates in China

**DOI:** 10.1371/journal.pone.0166069

**Published:** 2016-11-15

**Authors:** Ping Yu, Yuesong Pan, Yongjun Wang, Xianwei Wang, Liping Liu, Ruijun Ji, Xia Meng, Jing Jing, Xu Tong, Li Guo, Yilong Wang

**Affiliations:** 1 Department of Neurology, The Second Hospital, Hebei Medical University, Shijiazhuang, China; 2 Tiantan Clinical Trial and Research Center for Stroke, Department of Neurology, Beijing Tiantan Hospital, Capital Medical University, Beijing, China; 3 Beijing Key Laboratory of Translational Medicine for Cerebrovascular Disease, Beijing, China; 4 China National Clinical Research Center for Neurological Diseases, Beijing, China; 5 Vascular Neurology, Department of Neurology, Beijing Tiantan Hospital, Capital Medical University, Beijing, China; 6 Neuro-intensive Care Unit, Department of Neurology, Beijing Tiantan Hospital, Capital Medical University, Beijing, China; 7 Department of Neurology, Tangshan Gongren Hospital, Hebei Medical University, Tangshan, Hebei, China; Massachusetts General Hospital, UNITED STATES

## Abstract

**Background and Purpose:**

A case-mix adjustment model has been developed and externally validated, demonstrating promise. However, the model has not been thoroughly tested among populations in China. In our study, we evaluated the performance of the model in Chinese patients with acute stroke.

**Methods:**

The case-mix adjustment model A includes items on age, presence of atrial fibrillation on admission, National Institutes of Health Stroke Severity Scale (NIHSS) score on admission, and stroke type. Model B is similar to Model A but includes only the consciousness component of the NIHSS score. Both model A and B were evaluated to predict 30-day mortality rates in 13,948 patients with acute stroke from the China National Stroke Registry. The discrimination of the models was quantified by c-statistic. Calibration was assessed using Pearson’s correlation coefficient.

**Results:**

The c-statistic of model A in our external validation cohort was 0.80 (95% confidence interval, 0.79–0.82), and the c-statistic of model B was 0.82 (95% confidence interval, 0.81–0.84). Excellent calibration was reported in the two models with Pearson’s correlation coefficient (0.892 for model A, p<0.001; 0.927 for model B, p = 0.008).

**Conclusions:**

The case-mix adjustment model could be used to effectively predict 30-day mortality rates in Chinese patients with acute stroke.

## Introduction

Predicting 30-day stroke mortality rates in patients with acute stroke plays an important role in evaluating the prognosis of stroke patients [1,2]. Most previously published models have limited utility in clinical practice, as a result of being validated either in ischemic stroke [3,4] only or in hemorrhagic stroke [5,6] only, rather than in both. Although some models have been validated to accurately predict the mortality after ischemic stroke in Asian populations [7,8], they were not generalizable to both ischemic stroke and hemorrhagic stroke. As a result, these models cannot be used to compare the quality of care among different stroke treatment centers.

Case-mix adjustment models have been developed to meet the need of reporting case mix-adjusted mortality outcomes for stroke treatment services. However, most of these models included multiple variables, including the Oxfordshire community stroke project (OCSP) classification [9,10], plasma glucose levels [11], or those difficult to obtain, such as the GCS verbal score [12]. These limitations may explain why none of these case-mix adjustment models have been incorporated into routine clinical practice.

Recently, Benjamin D. Bray et al developed and validated a model to predict the risk of death for patients with acute stroke within 30 days after admission [13]. This model was derived from a large, prospective national registry of unselected cases of acute stroke in hospitals in England and Wales. The model was relatively simple and easy to implement. Two final models were eventually developed. Model A included age (<60, 60–69, 70–79, 80–89, and ≥90 years), presence of atrial fibrillation on admission, National Institutes of Health Stroke Severity Scale (NIHSS) score on admission, and stroke type (ischemic versus primary intracerebral hemorrhage). Model B was similar to Model A, but included only the consciousness component of the NIHSS score (NIHSS _1A) instead of all of the components comprising the NIHSS score [13]. However, there were few data on whether the model was suitable to use for Asian patients with acute stroke. Research has consistently shown that the incidence of stroke has been surging dramatically in low-to middle income countries. The average onset age for first stroke among individuals in China is approximately 10 years younger than among individuals in Western countries, and individuals in China have a higher percentage of hemorrhagic stroke[14]. Therefore, heterogeneity exists among stroke patients in Asian and Western countries. The aim of our study was to evaluate the accuracy of both model A and model B using data from the China National Stroke Registry (CNSR).

## Methods

### External validation cohort information

The CNSR is a prospective, nationwide hospital-based stroke registry created between September 2007 and August 2008. Eligible patients were enrolled if they were ≥18 years old and had ischemic stroke, transient ischemic attack, intracerebral hemorrhage, or subarachnoid hemorrhage within 14 days of the index event from 132 hospitals across China. The stroke events were confirmed by brain CT or MRI within 14 days after the onset of symptoms. The design, rationale and baseline characteristics of the CNSR have been reported elsewhere [15]. The registry was approved by the central Institutional Review Board at Beijing Tiantan Hospital. Informed written consent was obtained from all patients or their designated relatives. Authors had no access to identifying participant information during or after data collection. Our present study included only patients with acute ischemic stroke or primary intracerebral hemorrhagic stroke.

### Risk factors definition

The baseline severity of neurological impairment was evaluated by the National Institute of Health Stroke Scale (NIHSS) within 24 h after admission. These data were collected via an interview conducted by trained research coordinators. Other data, including patient demographics (e.g., gender, age) and vascular risk factors, were extracted from the medical records. Baseline vascular risk factors included history of hypertension (history of hypertension or antihypertensive drug use), stroke or TIA (defined as being confirmed in a medical chart), dyslipidemia (history of dyslipidemia or lipid-lowering drug use), diabetes mellitus (history of diabetes mellitus or hypoglycemic drug use), atrial fibrillation (history of atrial fibrillation confirmed by at least one electrocardiogram or presence of this type of arrhythmia during hospitalization) and history of coronary heart disease, current or previous smoking, etc.

### Outcome measures

The main outcome of interest was all-cause mortality rate within 30 days after admission, which was confirmed via telephone by trained research personnel at Beijing Tiantan Hospital.

### Statistical analyses

Age and NIHSS scores were reported as medians (interquartile range); all the other values were percentages. Significance testing of age and NIHSS was performed via t-test for continuous variables and the others were by χ 2 test for categorical variables. Discrimination of the case-mix adjustment model in our study was assessed by receiver operating curve analysis and estimation of the area under the receiver operator curve (c-statistic) and by calibration by plot of observed versus predicted mortality with 10 deciles of predicted risk.

The case-mix adjustment model on which our analysis was based has been published elsewhere [13]. The final two models (Model A and Model B) produced from Bray were both used directly and validated in our study. Comparison between the observed and predicted mortality was assessed by Pearson’s correlation coefficient. Given the large sample size used in our study, we did not use Hosmer and Lemeshow goodness-of-fit test, which is known to be sensitive to sample size, to assess calibration. All analyses above were conducted using SAS (version 9.4; SAS Institute, Cary, NC).

## Results

Among the 22,216 patients enrolled in the CNSR, there were 12,415 patients with ischemic stroke and 3,255 patients with primary intracerebral hemorrhagic stroke who consented to participate in follow-up. After excluding 1,722 patients without NIHSS_1A information, a total of 13,948 patients with complete 30-day mortality information were included in our study. Comparing the demographics of CNSR patients with stroke with those in the Sentinel Stroke National Audit Program (Table 1), CNSR patients were younger (median, 67 versus 77 years of age), more likely to be women (68.0% versus 50.2%), were more likely to have had hemorrhagic stroke (20.7% versus 10.5%), and had a lower mortality rate at 30 days (10.9% versus 13.2%). There were no clinically significant differences in baseline characteristics between patients included and those excluded; however, there was a slightly higher proportion of history of stroke or TIA and dyslipidemia among the patients included (Table 2).

**Table 1:** Characteristics of the model derivation and the external validation cohorts.

	Derivation	External Validation
**Dataset size (n)**	9000	13948
**Dataset Duration**	January to June 2013	2007–2008
**Source**	Patients with stroke included in Sentinel Stroke National Audit Program	Patients with stroke recruited to the China National Stroke Registry
**Age (median,IQR),y**	77 (67–85)	66 (56–74)
**Female (n,%)**	4518(50.2)	9343(68.0)
**Major Ethnicity**	White	Han
**Stroke type (n,%)**		
**Ischemic stroke**	8055(89.5)	11056(79.3)
**Primary Intracerebral Hemorrhagic stroke**	945(10.5)	2892(20.7)
**History of atrial fibrillation (n,%)**	1827(20.3)	879 (6.3)
**30-day mortality (%)**	13.2	10.9
**Median NIHSS on admission (IQR)**	4(2–10)	5(2–11)

Abbreviation: NIHSS, the National Institute of Health Stroke Scale. NIHSS was evaluated within 24h after admission to hospital.

**Table 2:** Characteristics between patients with NIHSS and those without NIHSS in CNSR.

Characteristics	NIHSS not recorded	NIHSS recorded	P value
**Sample size (n)**	1722	13948	
**Age, median (IQR), y**	65(55–74)	66(56–74)	0.01
**Male**	1071(62.20)	8582(61.53)	0.59
**History of:**			
**Atrial fibrillation**	93(5.40)	879(6.3)	0.14
**Previous stroke or TIA**	518(30.08)	4605(33.02)	0.01
**Coronary artery disease**	199(11.56)	1797(12.88)	0.12
**Diabetes mellitus**	309(18.21)	2617(18.80)	0.83
**Peripheral vascular disease**	13(0.75)	76(0.54)	0.27
**Hypertension**	1069(62.81)	8919(64.00)	0.51
**Dyslipidemia**	160(9.41)	1460(10.50)	0.00
**Atrial fibrillation in hospital**	119(6.91)	914(6.55)	0.57
**Smoking**	493(29.49)	3642(26.86)	0.06
**Dead in the hospital**	84(4.88)	565(4.05)	0.10

Abbreviation: CNSR, China National Stroke Registry. Medical history was defined on the basis of preexisting conditions, with the exclusion of conditions that were newly diagnosed during the hospital stay. Atrial fibrillation in hospital was defined according to the clinical manifestation and the findings on the electrocardiogram during the hospital stay. Age was reported as median (interquartile range); all the other values are percentages. Significance testing of age was by t- test (for continuous variables) and the others were by χ 2 test (for categorical variables).

**Fig 1:**
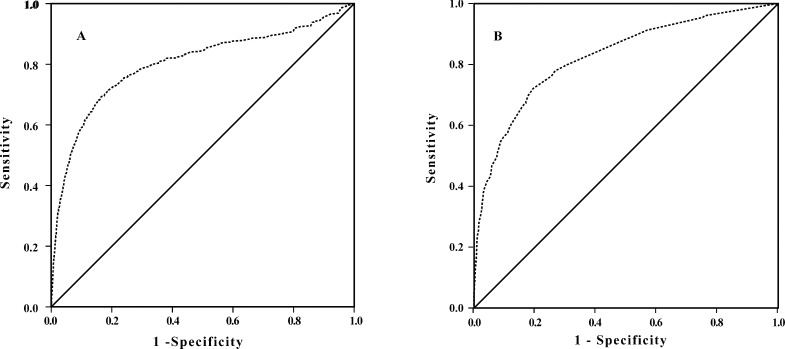
ROC curve analysis of the case-mix adjustment model predicting 30-day stroke mortality rates in the CNSR data set. Receiver operating characteristic (ROC) curve analysis of the case-mix adjustment stroke model A(A)and model B (B) in the China National Stroke Registry (CNSR) data set.

**Fig 2:**
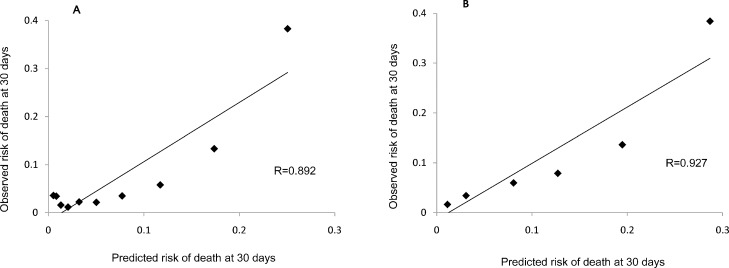
Observed vs predicted 30-day mortality in the external validation sample from the CNSR data set. Observed vs predicted 30-day mortality after admission of case-mix stroke for model A(A) and model B(B), according to 10 deciles of predicted risk in the external validation sample from the China National Stroke Registry (CNSR) data set. Overall, observed and expected mortality rates were highly correlated (Pearson’s correlation coefficient 0.892 in model A and 0.927 in model B), which indicates good calibration.

Based on the case-mix adjustment model A with full NIHSS score, the c-statistic of our external validation cohort was 0.80 (95% confidence interval, 0.79–0.82). For the case-mix adjustment model B, which included only the NIHSS_1A rather than the full NIHSS, the c-statistic of our external validation cohort was 0.82 (95% confidence interval, 0.81–0.84) (Fig 1). Excellent calibration was reported in the plot of observed versus predicted mortality rates in model A (Pearson correlation coefficient 0.892; P = 0.0005) and model B (Pearson correlation coefficient 0.927; P = 0.008) (Fig 2).

## Discussion

Our study showed that the two final simple models developed by Benjamin D. Bray et al could be used to predict the 30-day mortality rates in unselected type of stroke patients in the Chinese population. Model B, which included only the consciousness component of the NIHSS score, showed excellent performance, similar to that of Model A, suggesting the importance of the consciousness component of the physical examination. Thus, 30-day mortality might be evaluated based on Model B in stroke patients without complete NIHSS information, which is important in non-stroke centers.

Many studies have shown age to be a potent predictor of mortality after stroke [4,5,11,16–18], including both ischemic and hemorrhagic stroke. Atrial fibrillation is an important marker for cardioembolic stroke, which is associated with poor outcomes in thrombolytic therapy and with high mortality [19], probably due to the large infarction volume, a high risk of hemorrhage transformation, and lack of collateral blood supply. NIHSS score at admission, as a stroke severity marker, is a well-established and important predictor of mortality after stroke [7,20,21]. Although level of consciousness is only a component of the NIHSS score, it can forecast mortality after stroke both in the early phase and the long-term phase [22,23]. It can reflect the severity of stroke, possibly because severe loss of consciousness is often due to brain stem, large artery embolic or hemorrhagic stroke.

Many other case-mix adjustment models predicting mortality after stroke require complex assessments (Table 3), which limits their feasibility in routine stroke care [12,24]. The case-mix adjustment model in our study contains only items that could be easily extracted from the in-hospital medical records in China, and it is simple. Thus, it may be helpful for governments to monitor the stroke care services in different hospitals and for health insurers to analyze the cost-effectiveness of stroke care.

**Table 3:** Selected case-mix adjustment models predicting mortality after stroke and their variables.

	Variables included in model
**Belfast[25]**	Albert’s test score, leg function, conscious level, arm power, weighted mental score, non-specific ECG changes.
**Guy’s[26] (and G score[27])**	Limb paralysis, higher cerebral dysfunction + hemiparesis + hemianopia, drowsiness, age, unconscious at onset, uncomplicated hemiparesis.
**SSV[12]**	Age, living alone, independent pre-stroke, normal GCS verbal score, able to lift both arms, able to walk.
**SOAR[9] (and mSOAR[10])**	NIHSS, age, stroke type, OCSP, prestroke mRS.
**Uppsala[28]**	Conscious level, orientation, dysphasia, conjugate gaze palsy, facial weakness, arm power, Performance Disability Scale, reflexes, sensation.

There were limitations to this study. First, the centers participating in the CNSR might not be representative of the varying types of Chinese hospitals because the study recruited more centers in urban areas than in rural areas. Second, there might be selection bias due to the difference in history of stroke or dyslipidemia between the patients included and those excluded. Third, patients with undetermined stroke type were not included in the model derivation and validation; however, CT scanning is very common in China and is available 24 hours a day, 7 days a week in 99.2% of the hospitals, according to the China National Stroke Registry.

## Conclusions

In our study, we found that the case-mix adjustment model could be used to accurately predict 30-day mortality in Chinese patients with acute stroke.
